# Toward QoS Monitoring in IoT Edge Devices Driven Healthcare—A Systematic Literature Review

**DOI:** 10.3390/s23218885

**Published:** 2023-11-01

**Authors:** Muhammad Irfan Younas, Muhammad Jawed Iqbal, Abdul Aziz, Ali Hassan Sodhro

**Affiliations:** 1 Department of Computer System Engineering, Sukkur IBA University, Sukkur 65200, Pakistan; irfan.younas@iba-suk.edu.pk; 2 Institute of Space Science and Technology, University of Karachi, Karachi 75270, Pakistan; javiqbal@uok.edu.pk; 3 Department of Electrical Engineering, Sukkur IBA University, Sukkur 65200, Pakistan; aziz.memon@iba-suk.edu.pk; 4 Department of Computer Science, Kristianstad University, 29188 Kristianstad, Sweden

**Keywords:** quality of service, smart healthcare, Internet of Things (IoT), artificial intelligence (AI), machine learning, cloud computing

## Abstract

Smart healthcare is altering the delivery of healthcare by combining the benefits of IoT, mobile, and cloud computing. Cloud computing has tremendously helped the health industry connect healthcare facilities, caregivers, and patients for information sharing. The main drivers for implementing effective healthcare systems are low latency and faster response times. Thus, quick responses among healthcare organizations are important in general, but in an emergency, significant latency at different stakeholders might result in disastrous situations. Thus, cutting-edge approaches like edge computing and artificial intelligence (AI) can deal with such problems. A packet cannot be sent from one location to another unless the “quality of service” (QoS) specifications are met. The term QoS refers to how well a service works for users. QoS parameters like throughput, bandwidth, transmission delay, availability, jitter, latency, and packet loss are crucial in this regard. Our focus is on the individual devices present at different levels of the smart healthcare infrastructure and the QoS requirements of the healthcare system as a whole. The contribution of this paper is five-fold: first, a novel pre-SLR method for comprehensive keyword research on subject-related themes for mining pertinent research papers for quality SLR; second, SLR on QoS improvement in smart healthcare apps; third a review of several QoS techniques used in current smart healthcare apps; fourth, the examination of the most important QoS measures in contemporary smart healthcare apps; fifth, offering solutions to the problems encountered in delivering QoS in smart healthcare IoT applications to improve healthcare services.

## 1. Introduction

Health is one of the valuable assets for all human beings, and healthcare is the service which can help and guide in sustaining this asset. Currently, better and more economical healthcare services are needed more than ever due to the accelerated growth and increase in population and several diseases. Internet of Things (IoT), mobile, and cloud computing have greatly assisted the health sector in connecting health centers, caregivers, and patients for information exchange [1]. This arrangement, known as smart healthcare, is quite cost-effective for stakeholders for transmitting and receiving medical records. Thus, health devices generate large amounts of data and require processing as per the system target. The continuous expansion of smart healthcare systems has led to a massive growth in IoT healthcare devices, which are globally estimated to number more than 162 billion as of 2020 [2]. Therefore, considering the huge volume of data in an energy-constrained environment, contemporary communication architectures are becoming less effective. Similarly, prevailing computing methods are also failing to fulfill the performance expectations of smart healthcare applications. Furthermore, medical data is time-sensitive, and delayed medical data is of little help to caregivers (especially in emergencies). Low latency and better response time are the key enablers for realizing efficient healthcare systems. Therefore, swift responses among healthcare entities are of general significance, but in emergencies, high latency for various stakeholders can lead to catastrophic circumstances.

When it comes to healthcare services, low latency and quick response times are crucial for swift data access, which enables accurate diagnosis. The following few real-world scenarios highlight their significance:

- A patient in a remote area with few medical facilities can receive the care they need if the streaming videos between the doctor and patient work without any glitches. The doctor will be better able to evaluate the patient’s symptoms and make an accurate diagnosis.
- Low latency is advantageous for X-rays, MRIs, and other medical imaging since it allows for speedy loading for the doctor and several viewing angles for quick interpretation of the delivered reports.
- When a patient needs emergency care, it might be possible to save their life and guarantee that they receive the right care if a clinician has quick access to their medical records with no noticeable delays.

Cloud computing provides massive resources (computation and storage) to IoT-enabled healthcare devices but experiences high latency and slow response time due to being far from the end devices. Hence, to manage such situations, innovative techniques like edge computing and artificial intelligence can resolve these issues [3]. In edge computing, data are processed in edge devices located at the brink of the network [4]. This technique contributes to less latency and is more energy efficient. This enables edge-assisted IoT systems to deliver medical services on time. Moreover, combining these two technologies can lead the way to providing solutions to many challenging problems in healthcare systems.

Analysis of medical data can be greatly improved by employing AI techniques and can reduce the need for human intervention for decision-making. AI can predict diseases by investigating medical records and can suggest prevention or treatment of the predicted diseases to the patients. AI techniques need more computational power, so less resource-hungry AI techniques are needed in edge computing [5]. In edge computing, AI methods such as ML (machine learning) and DL (deep learning) are widely used for system training and learning. The combination of AI and edge computing known as edge intelligence is transforming smart healthcare applications. In edge intelligence, AI services and IoT data are divided into fragments and these subsets are deployed in different edge devices. Therefore, edge devices may contain complete or partial AI service/s or IoT data. Hence, services are transferred from the cloud servers to the edge-assisted IoT devices, providing AI and data storage closer to end-users [6]. At the same time, the IoT-based healthcare system consists of various devices with different requirements. These IoT devices pose several challenges, like higher battery lifetime requirements, interference from other devices, environmental signal attenuation, poor reliability due to more delay, etc.

In general, accurate diagnosis, prompt treatment, and excellent patient care define high-quality healthcare services. Technically, the smooth operation of medical monitors and equipment assures the patient that his or her information will smoothly and promptly reach the doctor’s computer. This will guarantee that the patient receives appropriate medical attention on time. This enhances the quality of healthcare service by improving response time and decreasing waiting between patients and doctors in state-of-the-art medical facilities as well as remotely. The quality of service parameters in the wireless link are of utmost priority, which in turn provides improved signal receptions, lowers packet loss ratios, and minimizes power drain. In addition to this, distributed AI services and IoT data present several QoS challenges, like battery lifetime, delay jitter, etc.

### 1.1. Review Motivation

With its cutting-edge connections between patients and healthcare professionals, smart healthcare is revolutionizing the industry. The core of the system is efficient communications. However, delivering QoS is challenging. There are not many publications that explore how to improve QoS in smart healthcare at various layers of the underlying network and services. Delay and energy efficiency concerns are of utmost importance for dependable communications within healthcare networks.

QoS is a set of technologies and techniques that are used to manage network resources and ensure that network traffic is delivered effectively. It enables you to prioritize different types of traffic so that important traffic gets through first and helps to prevent congestion on the network. QoS optimization is the process of configuring and fine-tuning these QoS settings to improve the overall performance and reliability of the network. This can involve setting priorities for different types of traffic, setting bandwidth limits, and using techniques such as traffic shaping and packet scheduling to manage the flow of traffic. QoS optimization is important in networks that carry a mix of different types of traffic, such as voice, video, and data, to ensure that the network performs optimally for all types of traffic.

**Figure 1:**
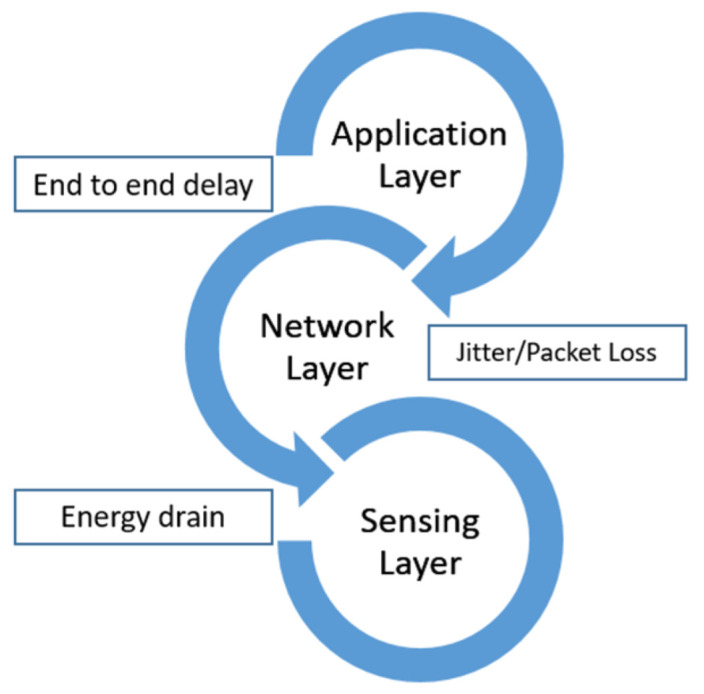
QoS requirements at different layers of smart healthcare applications [7].

QoS is the overall performance of the network service in terms of transmission delay, throughput, bit error rate, jitter, packet loss, energy drain, etc., from source to destination [7]. Currently, miniature size IoT enabled devices have greatly facilitated healthcare systems, but at the same time, their computationally hungry functions require more battery power and pose several challenges in edge AI-IoT-enabled healthcare systems. Our focus is on the QoS requirements of the healthcare system (as a whole) and as well as on the individual devices present at different levels of smart healthcare infrastructure, as shown in Figure 1.

### 1.2. Review Contributions

IoT, AI, cloud computing, machine learning, and other cutting-edge technologies are all part of the smart healthcare ecosystem. However, only a small number of articles on smart healthcare have considered QoS optimization. Our foremost contribution is to provide a thorough overview of all the key enabling technologies, covering everything from the fundamentals of smart healthcare to the most cutting-edge systems and services, such as delivering reliable levels of service for such applications. This article presents a systematic literature review (SLR). Along with it, the following list includes the areas on which our review focused:

1. An innovative method of describing pre-SLR activities that enables readers to judge whether an issue has undergone thorough research before examining it. To report on previous multidisciplinary investigations on this new paradigm, the study chose smart healthcare as the subject for conducting SLR. The mentefacto approach was used to produce keyword co-occurrence maps, and VOS viewer was used to extract pertinent research papers and analyze them. In MS Excel, thesaurus files were used to remove misspellings, synonyms, abbreviations, and elaborations. Section 2 elaborates on the entire strategy.
2. A systematic review of recent research on QoS optimization in smart healthcare applications is conducted, providing valuable insights for researchers and academics. The state of the art in QoS optimization for healthcare applications is revealed by this review, which incorporates articles, studies, datasets, and technology. Section 3 delineates the SLR.
3. By concentrating on how ML, cloud computing, and IoT are employed in smart healthcare, a study of the application of enabling technologies, smart healthcare, and QoS is offered. Secondary research analyzes primary studies using methods such as systematic mapping, reviews, and SLRs. The study highlights the lack of comprehensive studies and shortcomings in current methods utilized in smart healthcare applications. These investigations are presented in Section 4 and Section 5.
4. Smart healthcare applications encounter challenges with QoS optimization due to network congestion, interoperability problems, real-time demand, and cost limitations. Low latency is required by real-time requirements, network congestion produces unstable connectivity, and interoperability provides seamless system and equipment operation. Section 6 discusses and presents solutions to these issues.
5. Articles are chosen for their relatedness to cloud computing, machine learning, and IoT in the context of smart healthcare. Reviewing the literature and extracting QoS parameters, citation counts, and views, we analyzed in the result section how smart healthcare applications and service quality are related.

To our knowledge, no other SLR has been written with such a theme or concentration. Moreover, to validate our claim we conducted a topic-level pre-SLR search elaborated upon in Section 2. In Section 3, SLR is delineated. Preliminary concepts are presented in Section 4. Related work is highlighted in Section 5. Section 6 explains SLR results and analysis. Section 7 is about responses to posed research questions. Section 8 presents the challenges faced by SHAs and Section 9 elaborates recommendations and lessons learned. In the end, Section 10 concludes the SLR with future work.

## 2. Pre-Systematic Literature Review

Smart healthcare has received a lot of attention since the recent COVID-19 pandemic. In order to deliver smart healthcare services reliably, QoS methods and improvements should be considered. In an endeavor to advance the body of knowledge in science, we chose this subject for SLR to present recent multidisciplinary investigations that have been conducted on this new paradigm. We believe that readers would benefit from and be guided by this in determining if a particular issue has gotten a lot of research before taking any topic under consideration. None of the Scopus, IEEE, or Google Scholar databases produced any matches when the exact title of the SLR in double quotes was searched. In this part, an innovative and straightforward method for disclosing pre-SLR activity is discussed.

**Figure 2:**
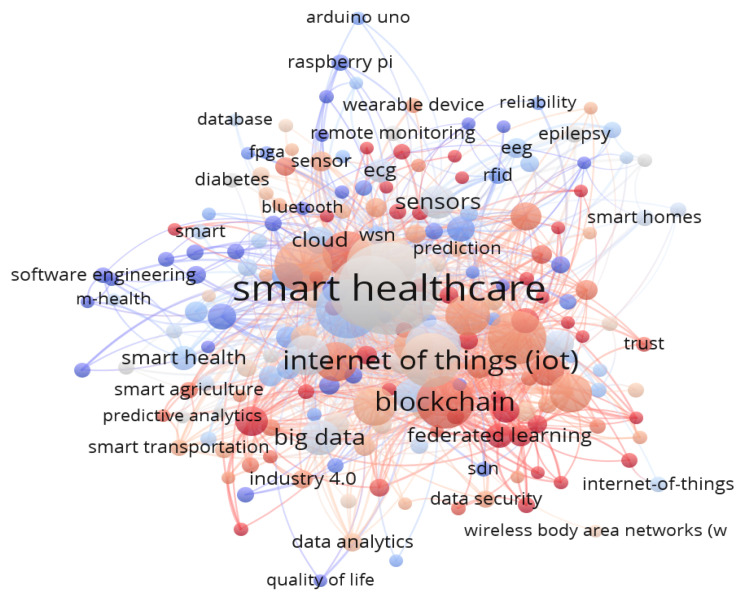
KCM of Scopus results as per keyword co-occurrence frequency [8].

To extract the most pertinent research papers for the SLR, the following steps were taken:

- A program called VOS viewer is used to create and display keyword co-occurrence maps (KCM), as shown in Figure 2 [8].
- Smart healthcare was used as a single search phrase to search the top internet databases (Scopus, IEEE, and Google Scholar) to create the keywords mentefacto, and the results were saved as CSV files.
- Thesaurus files were subsequently created in the MS Excel program to filter out keywords with various spellings, synonyms, abbreviations, and elaborations.
- To comprehend the knowledge structure of smart healthcare by looking at the keyword relationships in the literature, these files were input into a VOS viewer to draw a KCM [9].

**Figure 3:**
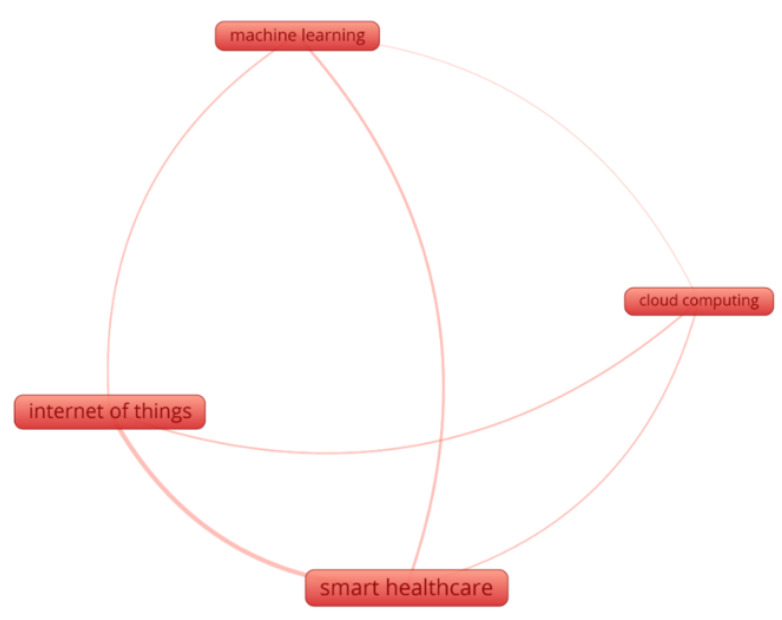
KCM of Scopus results after removing synonyms, abbreviations, and different spellings using thesaurus.

According to KCM, the top three keywords are “IoT”, “Cloud”, and “ML”, with “smart healthcare” appearing in Figure 3. Indeed, it is thanks to these technologies that smart healthcare is already a reality. The strength of the relationships reveals how frequently the terms are used together. All three technologies have several connections to smart healthcare, indicating potential uses for them there. Additionally, link strength analysis reveals that recent research in smart healthcare has emphasized IoT and ML over cloud computing, with the latter receiving less attention.

More connections exist between IoT and machine learning than between ML and cloud computing. This analysis shows that the combination of ML and IoT is more recent than the combination of ML with cloud computing. It is also likely that because the former is a relatively new concept, the scientific world is just now beginning to pay attention to it. The connections between IoT, ML, and cloud computing also give useful data, which are compiled in Table 1.

**Figure 4:**
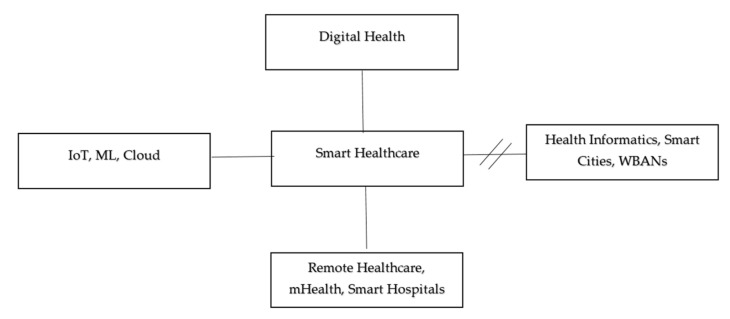
Keywords mentefacto indicates the hierarchy of included and excluded keywords [10].

The findings of the keyword-based search process were then taken into account while designing the keywords mentefacto as seen in Figure 4. As may be seen, a well-organized SLR is suggested by the hierarchy of searched terms. As a final step in pre-SLR, QoS synonyms, abbreviations, and elaborations were looked for in the filtered articles in web databases.

## 3. The Systematic Review Process

Systematic reviews offer an in-depth review of the most recent research on a specific subject. Similarly, we conducted this SLR to compile and explain the current trends in the field of smart healthcare, with an emphasis on QoS methods for delivering credible SHAs. To identify and classify the QoS optimization in smart healthcare applications, a systematic review of available and recent research is carried out. The researchers and academics would receive knowledge and insights from this synthesis of numerous recent and cutting-edge studies. To the best of our knowledge, no other SLR regarding QoS optimization in smart healthcare applications has been published. This research drew on numerous articles, studies, datasets, and different technologies used in this field.

### 3.1. Research Questions

Raising the proper questions makes review studies valuable and significant to the research community since they influence the entire SLR process. Review questions explain any changes or evolution in the SLR topic’s status and suggest if these changes should be accepted as part of the body of knowledge [10]. Questions were organized in a hierarchy of fundamental inquiries to ongoing research difficulties to provide readers a thorough understanding of the subject. Additionally, RQs were in line with the research objectives and the most up-to-date procedures and techniques being used in SHAs. The research topics that follow are intended to reveal the state of the art in QoS optimization for healthcare applications. The following research questions are answered in this review:

- Which fundamental enabling technologies are primarily used in today’s smart healthcare applications?
- What is the scope of QoS optimization in recent smart healthcare applications?
- Which QoS parameters are mostly employed in current smart healthcare applications?
- What are the current QoS aware technologies, methods, tools, and datasets employed in healthcare applications?
- What are the current QoS optimization challenges and solutions in smart healthcare applications (SHAs)?

### 3.2. Search Strategy

Various reputed research data sources were explored to extract the required results from the well-known digital libraries as enumerated below in Table 2. The research community highly regards IEEE Xplore and ScienceDirect for their relevant material, open access, and variety of publication types. Their availability of search capabilities and citation analysis data in numerous formats is the most significant aspect. Additionally, Google Scholar offers a vast number of search results, some of which even point to high-quality research papers that may not be accessible through other search engines. Each database offers a variety of logical tools that can be used to enhance search terms in an easy and efficient way.

**Figure 5:**
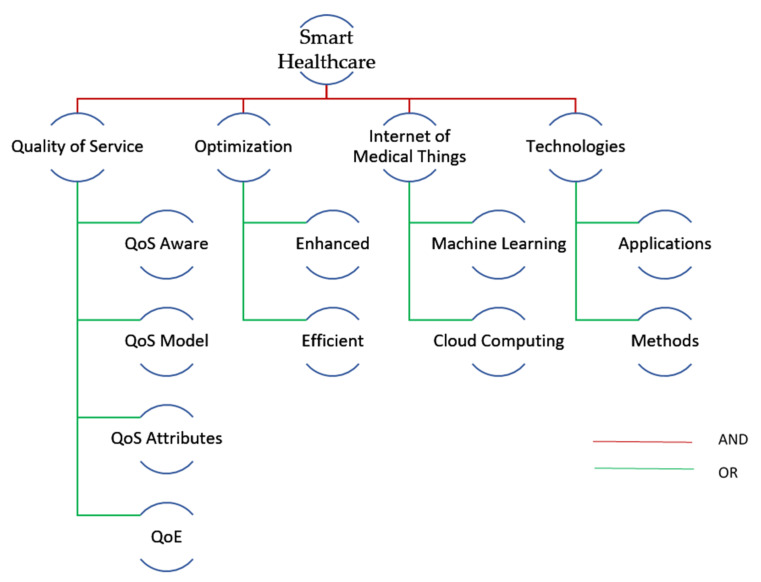
The search hierarchy shows the straightforward and mixed search terms that were used to look for the aforementioned data sources (using AND and OR).

The research questions were broken down into individual keywords, and an array of alternative spellings and synonyms was built. Furthermore, the query string was composed using Boolean logic operators of AND and OR. Figure 5 below shows the simple and sophisticated (using logical AND/OR operators) query strings, which were used to search the previously mentioned data sources [11].

The identified keywords were used to search online data sources and databases. It was discovered that longer keyword strings were not helping in determining individual keywords’ contributions. Keyword strings were kept under 5 to 6 words unless unavoidable. The below guidelines were followed:

- Advanced settings of databases were used to restrict search results or relevant papers and considered metadata comprised of TAK (title, abstract, and keywords).
- For every search pass, only the first 100 results were reviewed.
- Mostly recent publications (3 to 5 years old) were considered unless unavoidable.
- If highly important papers were not available because of any restrictions (e.g., paid or members only) then they were searched alternatively, such as on the author’s page.
- Reference management application was used to record and manage the references of the papers—web links, books, etc.

The searched research was selected as per the relevance of formulated research questions. This selection criteria were carried out iteratively. In the first iteration, only titles, abstracts, and keywords of the research were considered, and if required, the introduction and conclusion were also reviewed to determine the paper’s relevance with the research question [12]. Subsequently, the research methodology of the selected papers was reviewed to identify QoS parameters, network layers, technologies, and data sets employed in smart health applications. Moreover, other than relevance, the following inclusion criteria were considered:

- Research papers published in the last 3 to 5 years;
- Research papers in the English language only;
- Research papers published by academics only.

Likewise, the following exclusion standards were taken into account:

- Research published in dissertations, unpublished work, and editorial notes;
- Duplicates and research papers not available in the full text.

**Figure 6:**
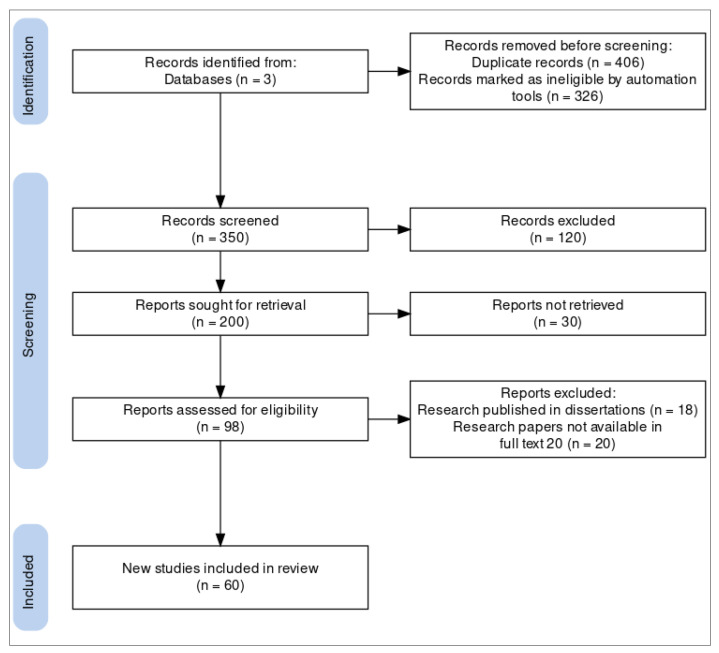
The SLR flow diagram outlines the selection process and selections taken at various stages of the systematic review as well as how the articles that were found were screened [13].

According to the specified search and quality assessment criteria, data were retrieved from the pertinent research papers as shown in Figure 6 [13]. Data analysis on QoS enhancement in smart health care applications. Furthermore, pertinent information relating to the research questions was recorded in our SLR.

## 4. Preliminary Concepts

This section provides a brief explanation of the notions of smart healthcare, enabling technologies, and QoS. It has been the objective of the research to explore further the present approaches of mixing various technologies to produce a high-quality SLR to describe the concepts and practices utilized in smart healthcare applications. According to this perspective, the goal of this research is to understand and examine the ways that current research is now producing, reporting, and applying the process of introducing and employing cutting-edge ICT technologies to provide smart healthcare services with a higher QoS. The top three technologies used in smart healthcare are IoT, cloud computing, and ML, as was discussed in the pre-SLR section. Further details on the applications of IoT, ML, and cloud computing are provided after an introduction to smart healthcare. The foundations of QoS, its metrics, and its application to smart healthcare are then investigated.

### 4.1. Smart Healthcare

**Figure 7:**
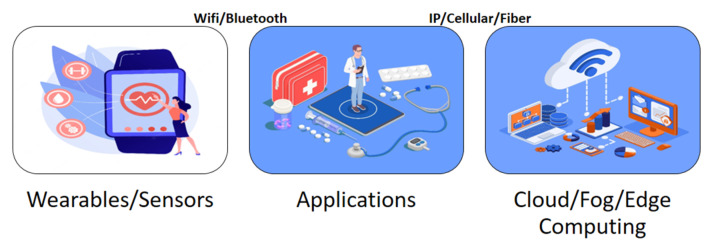
Model for a smart healthcare architecture that uses wearables, cloud, fog, and edge computing for healthcare applications [18].

Traditional healthcare cannot address all the needs of the population due to rapid population expansion. Hospital visits are challenging given the state of the planet, the most recent pandemic (COVID-19), the high expenditures, and the distances. To satisfy the demand for long-term care and remote medical monitoring, as well as to reduce the financial burden on patients by creating a digital healthcare system, it is essential to rely on technology that connects easily accessible medical resources and healthcare services. Smart healthcare is an IoT application that monitors, gathers, and analyzes medical data to provide online healthcare services. Smart healthcare could result in the development of a system of interconnected medical devices with sensors for the observation and treatment of patient health, as shown in Figure 7 [14]. Medical sensors are compact, sophisticated, reasonably priced, and light wearable sensor nodes used for health monitoring. They can be attached or connected to devices inside or outside the body. By effectively integrating these tiny devices with wireless technology, smart healthcare monitoring can be carried out using the Internet of Things [15]. By automatically interacting with them, these technologies alert users, physicians, and other healthcare providers, enabling them to deliver services effectively [16]. Given the growing trend towards smart cities, an effective smart healthcare system assures a healthy lifestyle for its citizens. Connectivity technologies are essential for developing smart healthcare applications. Smart healthcare apps are powered by cloud computing, machine learning protocols, and IoT. A patient record system that functions properly with the right sensing mechanisms and gathers structured and unstructured data for ML analysis is the technology and architecture of smart healthcare. Cloud computing makes it simple to access medical data from numerous sources, including test results and electronic medical records. Additionally, this framework offers analyses that allow medical professionals to compare how they perform to norms for high-quality treatment [17]. Smart healthcare integrates medical technology (sensors), the cloud, and humans (ML) to track, handle, and maintain patient records for continuing treatment and machine learning analysis.

The platforms and architectures used to achieve smart healthcare offer a wide range of features. Perception, networking, middleware, and application layers are the four levels that make up the architecture of smart healthcare apps. The three key components of the architectures and platforms utilized to achieve smart healthcare are configuration, organization, and framework. To build seamless healthcare service environments, a variety of important sensors and actuators with complementary applications are installed in heterogeneous computing grids as part of the configuration process. On the other side, the organization integrates the physical requirements for healthcare with the hierarchy of the design. Smart healthcare designs must be able to interface with several technologies—such as Wi-Fi, Bluetooth, LoRa, etc.—as medical sensors will be linked to one another by a body area network [18]. Organizations therefore look at the operational ideas and approaches used in network designs. Libraries and environments implementing the healthcare architecture are incorporated into a framework. Additionally, healthcare platforms can be divided into network, computing, and service platforms.

Network platforms connect several architectural types, whereas computing platforms are a nexus of more general ideas, such as database management, human–computer interface, machine learning techniques, etc. A “service platform” is a more sophisticated support layer that acts as a barrier between users and technology and could take the shape of call center personnel or automated chatbots [19]. As a result, services can range from straightforward warnings to context-sensitive ones. For instance, contacting an ambulance (based on geographic sensory data) for further paramedic assistance or reporting the number of miles traveled using a wearable sensor. Applications for smart healthcare include telesurgery, monitoring vital signs in intensive care units (ICUs), and fitness tracking in daily activities. Applications fall under two categories: emergency notification systems (ENS) and remote health monitoring (RHM) (ENS). Patients can obtain medical care for tracking symptoms, post-hospitalization care, etc., from home or in a remote geographic location. A recent pandemic (COVID-19) or endemic is just one example of a medical emergency that the ENS warns the general public or groups of individuals about. As a result of the use of mobile devices to facilitate medical follow-up, “m-health”, which utilizes and assesses health statistics, was also established [20].

### 4.2. Internet of Things

**Figure 8:**
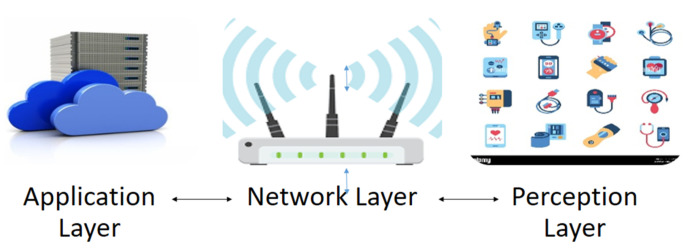
The IoT architecture consists of sensors and actuators for the perception layer, network layer protocols, and application layer components [25].

In a presentation describing how RFID may be used for supply chain management, Ashton, K. [21] introduced the phrase “Internet of Things”. The phrase “Internet of Things” today is used to refer to a network of devices and gadgets—including various kinds of sensors and actuators, mobile phones, and wearable technologies—that communicate with one another over the Internet [22]. These devices establish connections with servers so that information can be retrieved and transmitted between them, successfully providing essential services. This ground-breaking technology can be seen as a paradigm change in patient health-focused low-cost healthcare applications. The Internet of Things connects patients and clinicians in a setting that incorporates sensors, sophisticated algorithms, cloud interfaces, and communication interfaces. Technology advancements in sensor, RFID, and WSN networks support data collection infrastructure as shown in Figure 8. Advanced algorithms are then used to analyze the data [23]. Cloud services can be utilized to upload medical data and lessen the complexity of the scenario due to the resource limitations of IoT devices. Healthcare applications cannot effectively use the delays provided by cloud computing due to low latency, great dependability, and other criteria for healthcare. With real-time analysis and efficient decision-making tools, healthcare applications are run anywhere near users to overcome these limitations. To overcome these restrictions, fog, and edge computing run healthcare apps close to IoT devices with real-time analysis and efficient decision-making tools [24]. Despite all its advancements, the IoT continues to be in its infancy. Numerous issues—such as the heterogeneity of various devices, scalability, security, and privacy—are still being researched.

Transportation, smart cities, monitoring, healthcare, and other industries are among the applications covered by the IoT concept. Applications for the IoT can be used in the healthcare sector to monitor patients at the hospital or, more precisely, at home for elderly patients with chronic illnesses. This results in an earlier diagnosis, better treatment, lower healthcare costs, and a longer life expectancy [25].

### 4.3. Cloud Computing

**Figure 9:**
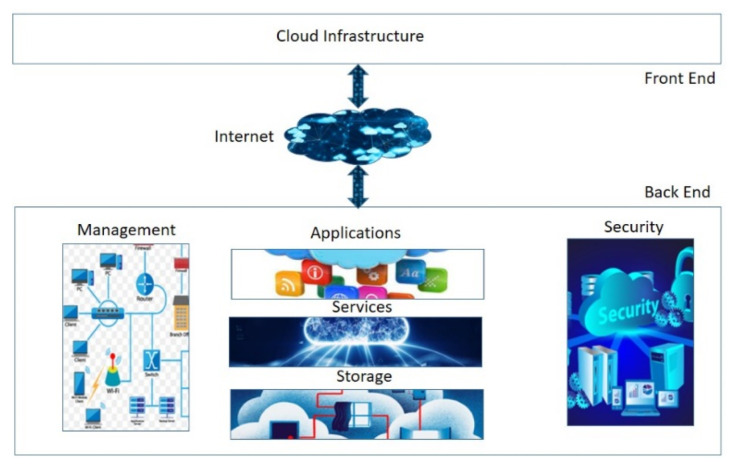
The front and back ends of a cloud computing system are two separate components. Over an intranet or via the internet, both sides of the connection can communicate with one another [26].

In cloud computing (CC) the provision of virtualized, on-demand computing, data storage, and networking capabilities via internet access via cloud services is completely customizable. The complexity and cost of CC resources vary depending on the desired capabilities and degree of application complexity for which they are used [26]. Compared to local computing resources, the cost, manageability, and flexibility of cloud computing resources have recently improved. Each data center in which cloud services are normally housed uses thousands of computers regularly. These systems must be able to scale up to extraordinarily high service demand levels while preserving reasonable processing times and low hardware and energy costs. This can enable interface simplicity that helps in processing widely accessible cloud data and offers a variety of services by linking multiple platforms. Infrastructure as a service (IaaS), platform as a service (PaaS), and software as a service are the three main services that make up CC services (SaaS). Companies may either buy or use CC services from cloud service providers, like Amazon, or they can build their own private clouds for their employees to use. Participatory community clouds are frequently used by research organizations and other pertinent organizations. In hybrid clouds, both private and public strategies are employed [27]. To assist better decision-making across a variety of application domains, including healthcare, applications enabled by CC solutions can extract highly important data. CC is one of the key enabling technologies for smart healthcare, which is made up of a variety of sensors, actuators, apps, and communication technologies, as shown in Figure 9.

Clinicians can provide services to clients wherever they may be thanks to cloud-based health services. Patients can use applications that operate on a variety of platforms, and their requests can be fulfilled automatically without the need for someone to handle them. The two cloud-based healthcare applications that are most frequently utilized are telemonitoring and electronic health records (EHR). EHR offer a standardized method for gathering electronic patient health data. Clinic settings may more easily share various sets of data, such as medical histories, vital signs, and medication, thanks to this standard platform. To provide healthcare remotely, medical telemonitoring employs information analysis and transmission methods. As indicated in [28], there have been various discussions regarding how cloud resources could be used more efficiently to provide high-quality healthcare services.

### 4.4. Fog/Edge Computing

**Figure 10:**
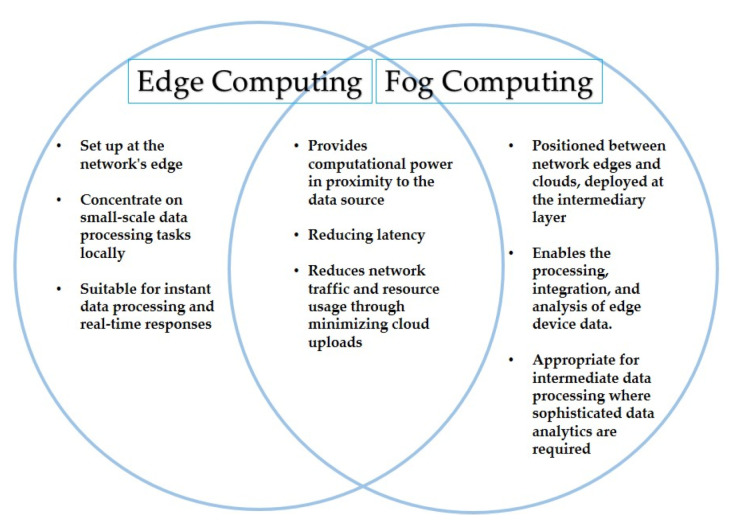
Venn diagrams show the key similarities and technical differences between the two computing paradigms.

Due to the numerous drawbacks of conventional centralized computing—such as single point failure, excessive latency, energy consumption, etc.—a more distributed and decentralized paradigm for cloud computing has emerged. Although CC does address several problems, such as single points of failure, it is still responsible for concerns with latency and energy use brought on by big uploads to the cloud servers. It became apparent that it would be best to upload only the data that required more processing to the cloud rather than the entire acquisition of data. It would be preferable to carry out operations that require lighter processing locally or very near to the user. Fog and edge computing are concepts that were inspired by this particular idea. This processing idea is shared by fog computing and edge computing. However, they differ technically as indicated by the Venn diagram in Figure 10.

Both paradigms are crucial in the context of SHAs for providing reliable services to the stakeholders. The application requirements can determine the specific choice. Fog computing, on the other hand, can be used for improved patient data analysis, such as in laboratory reports or prognosis, whilst edge computing can provide quick responses for patients at a distance from hospitals or doctors.

### 4.5. Machine Learning

**Figure 11:**
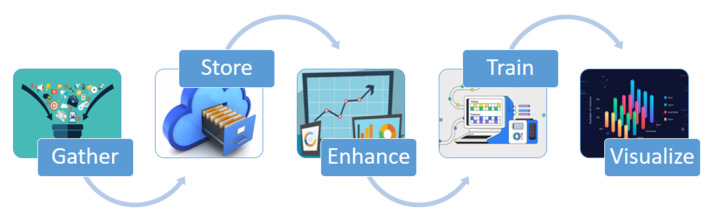
Machine learning is the process of using carefully crafted code to build systems that learn and evolve on their own [30].

Artificial intelligence, a new assistant for doctors, can help them with diagnostics and even prognoses. The ability to learn from experience can also make it easier to fully understand the conditions of the patients. The use of AI algorithms in SHAs allows for the creation of computer programs that acquire knowledge and grow as a result of experience rather than being explicitly programmed to make predictions or suggestions. Over the past few decades, technological advancements in computer power have made it possible for resource-intensive AI methods, like machine learning solutions, to be created. Machine learning programs use information for training, or data samples, to statistically develop a predictive model, as shown in Figure 11 [29]. Applications that are needed for sound decision-making employ this training to categorize things or make predictions. Smart healthcare applications use machine learning logic to process health data gathered from sensors and send it to the cloud for processing using machine learning algorithms. The received data are referred to as the testing data, and the outcomes are adequately emulated. After being emulated, the results will also be used for the training portion of the forthcoming testing data. The data obtained by the sensors are therefore viewed as testing data and, following processing, as training data for further medical assessments. Computational efficiency, detection precision, and robust implementation are the most important factors to consider when choosing machine learning algorithms for healthcare applications [30].

Typically, supervised learning, unsupervised learning, and reinforcement learning are used to categorize machine learning systems. The use of labeled datasets to train algorithms that accurately categorize or predict data or outcomes is the definition of supervised learning. By giving the algorithm examples of inputs and the anticipated outputs that they should produce, the goal is for the program to learn a general rule that connects inputs to outputs. Unsupervised learning refers to the algorithms used to find patterns in data sets, including data points that are neither categorized nor labeled. The learning algorithm does not receive labels; instead, it independently uncovers hidden patterns in the data and works as a feature extraction tool. An algorithm for reinforcement learning can perceive and comprehend its environment, act and learn from mistakes, and decide what actions intelligent agents should take to maximize the concept of cumulative reward in a particular environment. As it traverses its issue area, the learning algorithm interacts with a dynamic environment and receives feedback that could be compared to rewards, which it seeks to maximize [31]. A subset of machine learning known as “deep learning” algorithms may learn to carry out categorization tasks directly from images, text, or voice. They are supported by synthetic neural networks.

Vital sign data is gathered via sensors, which enables AI to spot trends in real-time. Triage systems powered by AI select cases according to their urgency, speeding up emergency response. By using the aforementioned AI techniques, large amounts of data produced by health sensors may be processed quickly and easily. This swift processing aids in prompt diagnosis, which decreases latency. Additionally, the use of AI-enabled remote monitoring devices, automation of the patient’s screening process, instant notifications, and predictive algorithms aid doctors in making timely decisions and substantially improve response time. Deep learning models are developed using a large amount of sample data and neural network designs with numerous layers to reach state-of-the-art accuracy [32]. Convolutional neural networks and deep belief networks are two examples of analytical deep learning models that provide computational intelligence solutions by studying sizable datasets in circumstances when shallow learning is unable to investigate the necessary meaningfulness of trends. These learning models are frequently used in precision medicine for disease diagnosis and therapeutic development processes [33]. The ML algorithms used to anticipate and classify health data exhibit an analytical pattern and provide results that are generally acceptable. AI and edge computing integration in smart healthcare enhances latency, efficiency, and tailored healthcare delivery, promoting a responsive, dependable, and effective system. They are regarded as a key enabling technology in this SLR due to the frequent use of categorization and prediction models in smart healthcare applications.

### 4.6. Quality of Service

**Figure 12:**
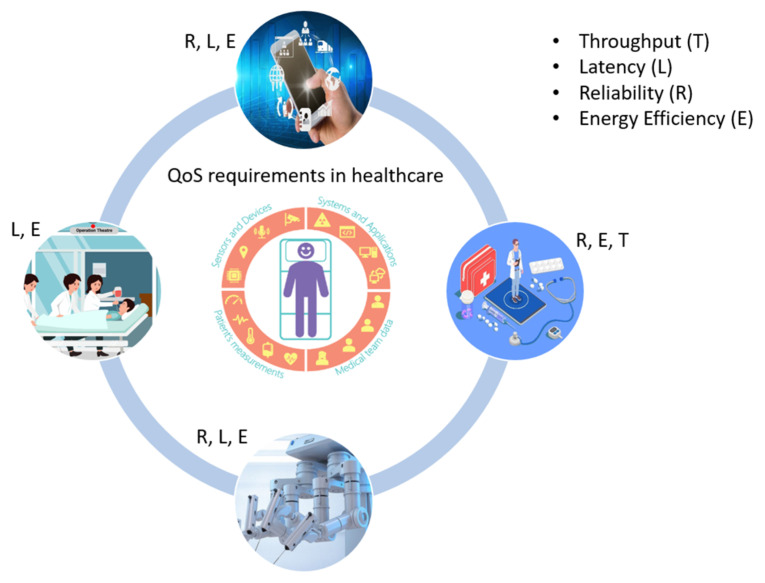
In terms of various requirements, different healthcare sectors demand QoS guarantees [34].

Given how delicate the subject of health is, accuracy and perfection are paramount. Because health facilities cannot accept network performance difficulties or downtime caused by QoS solutions that were earlier suitable, providing QoS is a crucial obligation for smart healthcare apps. Since the information is so important to the patient’s health, the system needs to quickly gather reliable data. The QoS requirements must be met when a packet is sent from one place to another as shown in Figure 12. The throughput, bandwidth, transmission delay, availability, jitter, latency, and packet loss metrics are among them, as pointed out by Sodhro in [34]. They are defined as:

- Throughput: within the time frame specified, data transmission traveled between two points.
- Bandwidth: the optimum rate at which data can move through a network.
- Delay: elapsed time for data traffic to reach its destination.
- Availability: ratio of network’s accessibility to inaccessibility by its users.
- Jitter: rate of change in data packets’ delays.
- Latency: the sum of the time it takes a data packet to travel from its source to its destination plus any computational delays.
- Packet loss: network issues preventing data packets from reaching their destinations.

A fundamental QoS model considers the overlay apps and the properties of the underlying network. These views need to be managed consistently because healthcare requirements are constantly changing. Based on their requirements, the network components are in charge of giving the apps a certain level of QoS. Applications can better track QoS metrics, such as the access network, battery life, client preferences, and delays.

The performance of a network is evaluated using QoS measures. The physical, link, network, transport, and application layers of the network stack are only a few of the layers where these parameters can be considered. Signal strength, signal-to-noise ratio, and bit error rate are examples of QoS parameters that can be applied at the physical layer. Packet loss rate, latency, and jitter are examples of QoS parameters at the link layer. Throughput, routing effectiveness, and congestion control are examples of QoS criteria at the network layer. Round-trip duration, retransmission rate, and error correction are examples of QoS parameters that can be used at the transport layer. Response time, availability, and reliability are examples of QoS factors that can be applied at the application layer. According to the needs and objectives of the network and applications, certain QoS factors may generally be more relevant or significant at different tiers of the network stack.

## 5. Related Work

**Figure 13:**
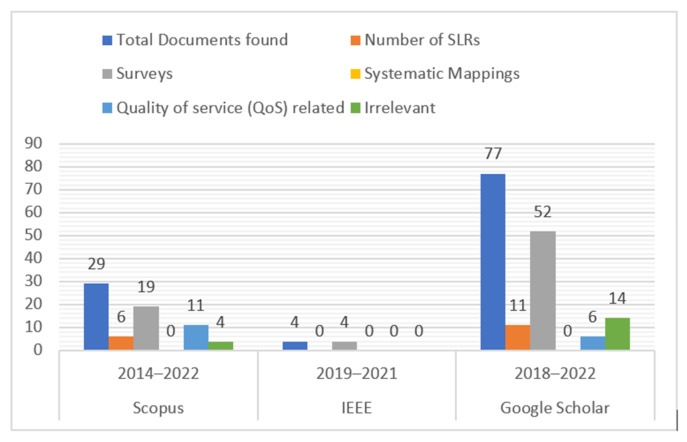
Analysis of prior studies on smart healthcare to identify recent review study methodologies and their emphasis on quality of service.

Secondary research examines prior studies—primary research—to direct future study planning and offer practical insights. Methodologies used in secondary research include systematic maps, reviews, and SLRs. The accompanying SLRs, surveys, and systematic mappings in smart healthcare applications are examined in this part to highlight the dearth of comprehensive evaluations and shortcomings of current methodologies. “Smart healthcare” was searched for in Scopus, IEEE, and Google Scholar (article titles only) to find related literature. For this part, only survey articles (SLRs, surveys, and systematic mappings) are taken into consideration. The results of the search are analyzed in Figure 13.

### 5.1. Systematic Literature Reviews/Surveys/Reviews

In this section, an overview of pertinent evaluations of smart healthcare is covered. These reviews fall within the categories of systematic mapping publications, surveys, and SLRs.

An SLR conducted by R. Dwivedi [35] focused on IoMT applications in smart healthcare systems and delved into detail regarding how IoMT works. As examples of relevant modern technologies, extended reality (XR) and its subsets, parallel processing techniques and their variations, data technologies, and 5G were cited. The limitations and uses of IoMT in the healthcare industry are then covered. Despite PRISMA guidelines being followed, no research questions are mentioned, and no QoS-related issues or challenges are discussed.

The analytics from recent studies on IoT applications in healthcare are provided in [36] by the authors. This SLR has tackled issues and obstacles relating to system, user, cost, and data to offer IoT-based healthcare services. The writers gave the SLR title their entire attention, but they failed to adequately handle QoS, one of the toughest problems in healthcare. The article’s [37] major goal was to make clear the standards and factors to be considered when integrating IoT into healthcare systems. In addition to a comprehensive SLR, questionnaires and surveys were also conducted to investigate several IoT-based healthcare solutions. During COVID-19, a lot of focus was also placed on the IoT and how IoT-based telemedicine reduced the risk of infection transmission. There was also a preliminary breakdown of the difficulties with money and privacy in telehealth. A detailed SLR was conducted as part of another study [38] that concentrated on IoT and AI-based E-cardiac care. Reporting on academic research that uses IoT and AI to diagnose various heart irregularities may help people understand cardiovascular issues. The E-Cardiac architecture and core formalized RQs were fully addressed. Considerable amounts of research was reviewed, outlining both the advantages and limitations of AI- and IoT-based cardiology.

In [39], discussion is confined to spotting weaknesses in edge-enabled smart healthcare’s blockchain-based security concerns. Access control, node security, and data transmission security have all been mentioned in recent literature as blockchain-based security topics. The authors concluded that it will only be feasible to detect and address any potential operational issues and hidden risks by putting edge-enabled smart healthcare systems into use in the real world. In a survey [40] on blockchain technology, many approaches for sharing and obtaining access to health data among healthcare organizations were covered. According to a substantial blockchain application addressing patient-based ownership of their own health data that was offered, patients would own their data and be able to select who had access to it.

A list of various smart health monitoring (SHM) frameworks that leverage ML and other contemporary technologies has been created by the study [41]. The authors have talked extensively about the use of various ML approaches in SHM as well as cloud and fog computing. Network QoS was also addressed, and fog/edge computing was offered as a remedy to reduce network delay and reaction time in healthcare applications. Further suggestions for improving the QoS of fog/edge computing-based healthcare systems included fault-tolerant and multi-tiered architecture.

The TOE (technology–organization–environment) paradigm was used by Renukappa [42] to analyze why the health sector failed to implement smart strategies. The paper describes the difficulties in adopting smart technologies in healthcare. According to the results, organizational difficulties are the biggest barrier to implementing clever strategies. The two most challenging issues in this regard are general planning and a lack of cultural transformation. One of the key technical issues is cost and security. End users’ willingness to transition to smart healthcare applications is the key tenet in implementing smart strategies for healthcare in environmental issues. Every difficulty is covered in detail.

### 5.2. Comparison

The authors of the aforementioned papers have tried to include as many models, frameworks, and technologies as they can in their publications. However, their literature evaluations are only concerned with general characteristics of enabling technology in smart healthcare. It was discovered that just one review article [41] was comparable to our review paper, but QoS was not stressed, and the same coverage of too many technologies was also noted.

The scope of QoS optimization has increased in current smart healthcare applications to cover several important areas, such as monitoring and control in real-time: Smart healthcare applications use a range of sensors and equipment to keep tabs on patients. For these devices to operate consistently and properly, as well as for the timely and accurate transmission of the data they collect, QoS optimization is crucial [43]. Patients can receive care remotely through telemedicine and other forms of remote monitoring thanks to smart healthcare applications. QoS optimization is crucial for ensuring that patient data are transferred swiftly and accurately and that video and audio communications are clear and have low latency. Predictive analytics and machine learning: smart healthcare applications evaluate patient data and spot potential health hazards using predictive analytics and machine learning. The speedy and accurate completion of these studies and the accessibility of the data for healthcare practitioners are both dependent on QoS optimization [44]. Smart healthcare applications must be able to communicate with a variety of tools, platforms, and systems. This is known as interoperability. The effective communication of these systems and the accuracy and dependability of the data they exchange depend on QoS improvement. Security and privacy: reliable healthcare applications must ensure that patient information is not accessed by unauthorized individuals and is only utilized for the purposes for which it was collected. QoS optimization is essential for the security of the systems and the data [45]. In general, QoS optimization is a crucial component of smart healthcare applications because it guarantees that patients receive the best care possible and that healthcare professionals have the information, they need to make wise decisions. The analysis of the survey articles and SLRs mentioned above is summarized in Table 3.

## 6. Analysis of SLR Results

This section presents comprehensive SLR findings from our research on QoS enhancement in SHAs. As a result, 15 highly relevant technical papers on QoS optimization and energy efficiency were found, with an emphasis on SHAs.

### 6.1. Paper Distribution

**Figure 14:**
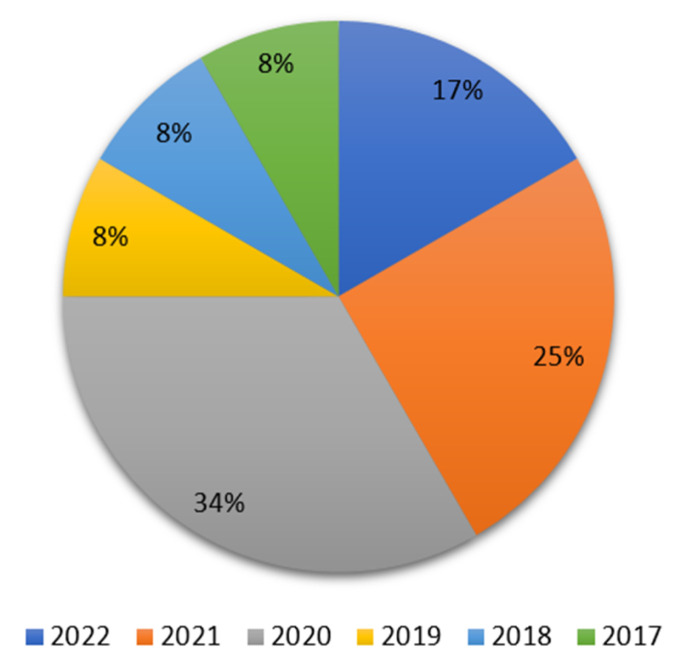
Article distribution according to year of publication.

The papers were categorized based on the study’s goals and the year they were published as shown in Figure 14. Additionally, a citation analysis is performed to determine the current rate of research effort in the QoS of SHAs.

The pie chart illustrates the quantity of recently released filtered research publications. The fact that more than half of the articles were released in 2020 and 2021 shows how much the research community has recently been interested in QoS-related issues in SHAs.

**Figure 15:**
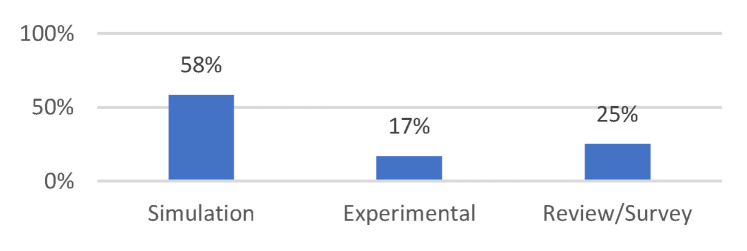
Distribution of papers according to research methods.

Figure 15 displays the distribution of research articles by study methodology. Studies that used simulations predominated, followed by publications and surveys that had an experimental focus. Simulator-based papers were those that simply employed simulators, whereas experimental-based papers included some or all hardware interfacing. Finding review or survey articles proved challenging. This distribution demonstrates the QoS research direction for the smart healthcare system. Furthermore, the dearth of review publications points to the necessity of SLR in this area of research.

**Figure 16:**
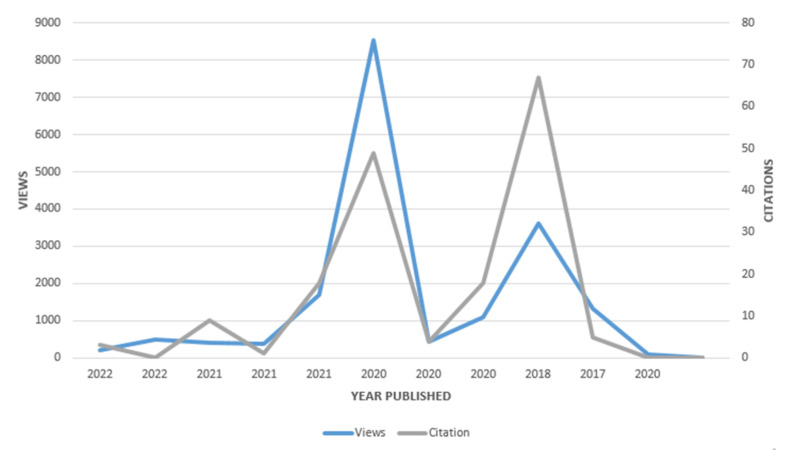
This pattern shows that QoS issues in smart healthcare are receiving increased attention from the research community [46,47,48,49,50,51,52,53,54,55,56].

A citation analysis was also conducted, as shown in Figure 16, to evaluate the impact of selected works on the research community and body of knowledge. Although it was not the most popular piece, the most-cited article was published in 2018. On the other hand, the most well-liked piece was released later, in 2020, and garnered fewer citations. The article that had the least number of views and citations was the one that appeared first, in 2017. As indicated by increasing views but relatively limited published research output and consequently fewer citations, this trend shows that the research community has begun to pay greater attention to QoS issues in smart healthcare.

### 6.2. Golden Papers’ Synopses

These articles were also selected due to their strong connections to IoT, cloud computing, and machine learning, the three main pillars of smart healthcare.

The key themes of the papers are summarized and outlined. The literature reviewed below explores the connections between smart healthcare applications and quality of service. To show how influential each publication is in the subject of smart healthcare, QoS parameters, citation counts, and views are extracted. Simulation tools and datasets are also discussed to evaluate current trends in simulation software in the research community.

In [46], service latency is discussed at the network layer, with an emphasis on 5G networks, to meet the demands of service requesters (SRs). Resource allocation schemes are compared to determine the tradeoff between service satisfaction and revenue.

Specifically, a distributed network selection technique is presented for the heterogeneous wireless network environment [47]. The network selection problem has been characterized as an optimization problem to minimize the transmission energy consumption, cost, latency, and distortion. Network selection problems are addressed through the development of optimization strategies to obtain the best network possible within the constraints of resources and latency. The reward and convergence of the objective function are used to assess the method’s effectiveness.

The positive effects of edge computing for smart alternatives for distributed computation as well as the evaluation of smart IoT medical sensing were underlined in this paper [48] by the authors. This means that by fully or partially educating edge intelligent nodes at the edge of the network level, system latency can be decreased. It is possible to spread additional processing for computationally intensive applications throughout edge- as well as fog-based nodes or perform it in the cloud. Such methods offer considerable advantages in a situation where prevention and the early detection of symptoms are the primary considerations.

In a CR-WBAN analytical model based on EH, a primary sensor network and a secondary sensor network coexist [49]. The authors test the hypothesized WBAN of the CR system’s throughput performance under the specified parameters using the TSC (time-switching cooperation) and PSC (power-splitting cooperation) protocols. For the considered network, they studied the performance of two EH-based spectrum-sharing protocols, called TSC and PSC protocols, in terms of OP, throughput, and power efficiency over log-normal-distributed fading channels.

A new job-offloading approach has been presented [50] to decide whether to offload a job to a certain fog node or to a cloud server at a specific time. When there are no direct connections between IoT nodes and cloud servers, IoT nodes may occasionally offload a task to fog nodes first, and fog nodes may subsequently offload the activity to the cloud. To resolve the issue, a Markov decision process (MDP) is employed. The suggested MDP takes into account two decision-makers: IoT users can decide which fog nodes to assign their work to, and fog nodes can decide whether to split up specific jobs between themselves or to cloud servers to preserve the task balance. The simulation results show that the suggested methodology outperforms competing strategies in terms of minimizing delays, doing more tasks, and distributing the workload.

In this study [51], the confident information coverage hole repairing known as the CICHR problem was solved using the algorithms confident information coverage hole predicting abbreviated as CICHP, CEER, and DEER. The CIC (confident information coverage) concept served as an inspiration for these algorithms. The CEER and DEER algorithms have outperformed C-CICHH and D-CICHH, respectively, in terms of MSD, moving energy consumption, residual energy, and coverage ratio in the LS-HWSN based on the prior knowledge of confident information coverage hole that was acquired using the CICHP method. In comparison to C-CICHH and D-CICHH, simulation results show that the suggested algorithms can provide higher network QoS, a longer network lifetime, and reduced time complexity.

Feng [52] proposed a hierarchical approach called “green communication” for patient monitoring. The energy consumption of each device throughout each of its several states—idle, sleep, awake, and active—is modeled by this methodology. The base station chooses the cluster heads. A centralized routing system chooses the cluster heads at the base station level. The cluster heads no longer require join-request messages from the health monitoring instruments for the clustered to gather. As a result, they use less energy, which increases the network’s lifespan. Experimental outcomes demonstrate the effectiveness of our strategy.

The implementation of CoT in the context of smart healthcare is examined in this study [53], along with CoT designs and platforms. It is still necessary to find effective ways to achieve energy efficiency in data processing and transmission. The CoT architectures, platforms, and their use in healthcare were surveyed in this research. Instead of focusing on more general recommendations, it largely studied the energy efficiency problems in depth with the more pertinent proposals.

In this work [54], a rapid and dependable cloud resource allocation model for body sensor devices to ensure QoS for healthcare applications utilizing smart homes is developed. The suggested resource allocation strategy used Agent-Based Modeling (ABM) and the ABM application NetLogo. Numerous sensors are used by the CABAN platform to collect physiological data. Wearables, mobile devices, cloud platforms, participants, and display terminals make up the system’s five core components. It analyzes and saves the data via gateways and the cloud. Utilizing four different sorts of entities—turtles (moving entities), patches (stationary agents), connections, and the listener—provides a context for replicating natural processes. The evaluation is carried out by determining the algorithm’s execution time, which determines how resources are distributed based on input data. The findings show that agent-based modeling (ABM) can be used to address complicated challenges.

The proposed grey filter Bayesian CNN method [55] is introduced to reduce communication overhead besides response time. In comparison to recent efforts, the suggested GFB-CNN method shortens the end-to-end response time by only storing the best human activity using the Bayesian logistic sigmoid activation function. About the various numbers of instances, the performance of the proposed GFB-CNN approach is assessed in terms of communication time, overhead, and accuracy, and it is then contrasted with two previous works. The simulation results show that, when compared to cutting-edge works, the suggested GFB-CNN technique performs better. The proposed FB-CNN method, however, neglects to take security and privacy into account while analyzing medical data.

In [57], static sensors are installed to watch over sleeping patients, while mobile sensors track moving patients to collect data and store it on cloud servers. The proposed SHM consists of the SSOA, BSN, cloud services, and data management domains. An SSOA upholds security and QoS while engaging in activities. Knowledge-based repositories, cloud servers, and semantic information extraction in the data management area are all able to be accessed and exchanged by those with authorization. The mobile sensor recruitment and selection phase assists in obtaining sensors from other clusters for the cluster that lacks enough sensors—first from a neighboring area, then from a nonadjacent area—to improve throughput and reduce latency.

To solve the COVID-19 diagnosis problem effectively while maintaining patient privacy, this paper [58] offers insights into how edge computing and machine learning technologies might be used. By enabling remote healthcare units to take advantage of the collaborative learning paradigm without revealing local data, this paper advances the field of research. To analyze visual input intelligently at the edge, a multi-modal ML model that can recognize COVID-19 in both X-ray and ultrasound imagery is trained using a collaborative learning framework based on clustered federated learning (CFL). In comparison to conventional FL (such as X-ray and ultrasound images), it has been discovered that CFL can handle the divergence in data distribution from numerous sources better while still being able to meet strict security, privacy, and quality of service criteria (low latency).

To reduce network congestion, the suggested approach employed [59] a priority-based data routing strategy. In terms of the percentage of correctly received packets, average throughput, and average hop-by-hop delay, the suggested congestion management mechanism for healthcare-focused IoT networks is assessed and contrasted with the current renowned TARA methods in the literature. These findings suggest that in real IoT-based healthcare studies, our proposed strategy performs better than the current congestion control approaches. The presented method reduces the network’s overall energy use and increases the QoS. The study papers mentioned above were carefully examined, and the answers to the research questions are given below.

## 7. Responses to Research Questions

The answers to research questions are the main goal of this SLR. A distinct strategy was used to respond to the first two questions because they are frequently asked and appear in many review articles. Our special edition is that we initially responded to this query by earlier (pre-2018) research publications. Subsequently, we answered these queries again after completing SLR and determined the present (2018–2022) research trends in this regard.

### 7.1. Response to Research Question 1

As mentioned in the pre-SLR section, the same approach was adopted to search the literature published before the year 2018, and several key enabling technologies were found that were employed in smart healthcare applications a brief overview of those technologies is given below.

Internet of Things technology permits for the integration of medical devices, such as wearables and sensors, with healthcare systems to enable remote monitoring and communication [60]. It is covered more thoroughly in Section 4 in considerable detail. Big data in smart healthcare refers to the use of large and complex data sets to improve healthcare delivery, medical research, and public health. Smart healthcare systems generate and collect a vast amount of data from various sources such as electronic health records, medical devices, and wearables. By using advanced analytics and machine learning techniques on this data, healthcare professionals can gain insights that can improve patient outcomes and reduce costs. For example, big data can be used to identify patterns in patient data that indicate a potential health problem, such as a disease outbreak or an adverse drug reaction. It can also be used to optimize treatment plans and improve population health management. In medical research, big data can be used to identify new drug targets and biomarkers and to accelerate the discovery of new therapies. However, Big data in healthcare also poses some challenges, such as data privacy and security, data integration, and data governance. It is important to ensure that data is collected, stored, and analyzed in compliance with relevant regulations and guidelines for patient data privacy and security [61]. Large volumes of medical data can be stored, managed, and processed thanks to cloud computing. It also allows for remote access to health information and facilitates communication and collaboration among healthcare providers [62]. As mentioned and discussed in the pre-SLR section, the current research focus in smart healthcare is still very similar, but artificial intelligence and more specifically machine learning are quite prominent, along with IoT and cloud computing.

### 7.2. Response to Research Question 2

QoS is an important aspect of healthcare applications as it ensures that the system can provide a certain level of performance for real-time and mission-critical services. As of pre-2018 publications, QoS in healthcare applications was focused on medical imaging, telemedicine, electronic health records (EHR), and medical devices. In general, QoS in healthcare applications is essential for making sure that patients receive the most effective treatment and that healthcare professionals have the information they need to make wise decisions. Currently, research publications are more focused on network performance, data volume, data complexity, and system performance. Optimizing QoS in smart healthcare applications typically involves identifying and addressing the factors that are causing delays or other issues and implementing strategies to boost the general performance of the communication and data transfer processes. This may include things like optimizing network performance, minimizing data volume and complexity, and improving system performance.

### 7.3. Response to Research Question 3

The fact that SHAs are a superset of IoT, cloud, fog, edge, ML, and other technologies means that they inherit all the well-known characteristics of QoS as well as its difficulties and problems, including delay, latency, energy consumption, and other metrics. Specific QoS metrics have been considered crucial considerations in this domain because of the dynamic nature and strict requirements of smart healthcare systems. In emergency treatment, for instance, patient data must be provided and evaluated swiftly. Even though obtaining QoS in SHA applications is difficult owing to several limitations, it is the most desirable prospect in SHA applications. It is possible to fully utilize the QoS assessment of the technology and get beyond its challenges because SHAs have many uses. We investigated filtered articles in depth to identify the simulation models, QoS metrics, and sensors in SHAs, as shown in Table 4.

Currently, SHAs are experiencing difficulties with latency, delay, and energy consumption. Because of this, many QoS metrics for SHAs concentrated on ways to lower total energy consumption and boost network throughput while simultaneously prolonging network lifetime and assuring more dependable communication by lowering congestion and collision. We investigate metrics like end-to-end delay, latency, throughput, response time (RT), and energy consumption (EC) because we are primarily interested in network performance. The amount of time a data packet needs to traverse from its origin to its final location is referred to as network latency. The performance and efficiency of medical equipment and systems can be significantly impacted by delays in intelligent healthcare systems [63]. For example, in telemedicine applications, where patients and healthcare providers communicate and interact remotely through video and audio, high latency can result in delays and poor audio and video quality, which can affect quality of care. In remote patient monitoring applications, high latency can also affect the accuracy and timeliness of collected data as well as the ability to respond promptly to alerts and alarms. In general, smart healthcare systems need to have low latency to ensure that medical devices and systems function effectively, and that the quality of care is not compromised. To minimize latency, it is important to optimize the network infrastructure and design, as well as the protocols and algorithms used to transmit data.

**Figure 17:**
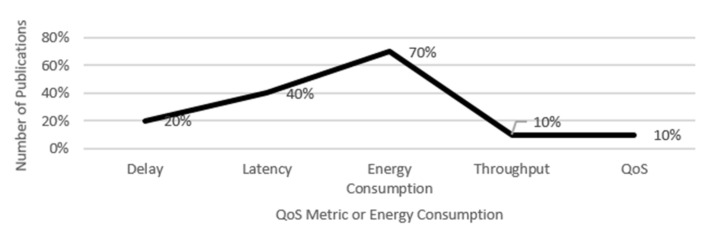
The QoS parameters related to SHAs that have received the most research include delay, latency, and energy consumption.

Moreover, datasets and challenges in adopting smart healthcare are also analyzed. The classification of researched articles is summarized in Figure 17 in terms of the QoS parameters employed over the enabling technologies for developing SHAs that are QoS aware [64]. Different models are applied to analyze QoS factors for efficient smart healthcare applications. Delay, latency, and energy consumption are the QoS metrics that have been the subject of the most research on SHAs, as is clear from the classification above. Furthermore, the top technologies used in SHAs are 5G, IoT, and cloud/fog/edge computing.

### 7.4. Response to Research Question 4

There are many QoS-aware technologies which are employed in healthcare applications, such as network virtualization and resource allocation. In [46], resource allocation is used to maximize SPs’ income while considering SR priority and interference in the 5G network. Using cognitive radio technology, A. K. Shukla [49] primarily focused on maximizing QoS through intelligent spectrum sharing, allowing different sensor nodes in the network infrastructure to coexist without jeopardizing QoS standards. For on-demand access to a shared collection of resources such as networks, servers, storage, and services to maintain QoS requirements, authors in [53] used the cloud computing concept. In [54], BANs are integrated with CC to fulfill the QoS requirements of caregivers. Traffic management regulates the flow of data in the network to ensure that critical traffic is delivered promptly and to prevent network congestion. Traffic management in healthcare refers to the process of controlling and directing the flow of patients, staff, and resources within a healthcare facility. This can include scheduling appointments, managing patient flow in clinics and hospitals, and coordinating the movement of medical equipment and supplies. The goal of traffic management is to improve the efficiency and effectiveness of healthcare delivery, as well as to enhance patient satisfaction and safety. This can be achieved with technology such as electronic medical records, computerized appointment scheduling systems, and real-time location tracking systems [65].

To handle the data traffic of healthcare applications and achieve the necessary QoS levels, researchers used a variety of routing algorithms in [51,59]. Quality of experience (QoE) measurement involves using specialized tools and techniques to measure the perceived quality of a healthcare service, including factors such as latency, reliability, and usability. In healthcare, QoE measurement can be used to evaluate the effectiveness of telemedicine systems, electronic health records, and other digital health tools. This can assist developers and healthcare practitioners in identifying problem areas and making the required corrections to improve user satisfaction [66]. In [46,51], QoE is addressed along with QoS to measure the technical aspects as well as the user’s perception of the service. Machine learning involves using machine learning algorithms to analyze and optimize QoS in healthcare applications, such as by predicting and preventing network congestion. Some of the most promising areas of research in machine learning for healthcare include [67] Computer-aided diagnosis, in which machine learning algorithms are used to analyze medical images to identify signs of disease. Predictive analytics uses machine learning algorithms to predict future health outcomes based on a patient’s medical history and other factors. This involves using data and analytics to predict and prevent issues that could affect QoS, such as network congestion or device failures [68]. Natural language processing (NLP) uses machine learning algorithms to extract information from unstructured data, such as electronic medical records. Currently, various ML algorithms and models are used in SHAs from underlying network selection to disease detection and even prognosis.

Methods that are quality-of-service-aware guarantee QoS while giving end users effective medical treatment. Research articles review a variety of methodologies, and they choose the most recent and widely used methodologies from the reviewed research publications. The prioritization method involves assigning higher priority to critical traffic, such as real-time medical data, to ensure that it is delivered promptly. Bandwidth allocation reserves a certain amount of bandwidth for critical applications and devices to ensure that they have the resources they need to function effectively. To ensure that vital traffic is delivered quickly and to avoid network congestion, traffic shaping modulates the data flow throughout the network. Error correction uses techniques such as forward error correction (FEC) to detect and correct errors in transmitted data to ensure that it is received correctly. Load balancing distributes traffic across multiple network resources to ensure that no single resource becomes overloaded and to improve overall system performance.

The electronic medical records (EMR) dataset [69] contains electronic medical records for many patients. It can be used to evaluate and optimize QoS in healthcare applications, such as by analyzing the effect of network latency on the precision and timeliness of data collection and analysis. Clinical trial datasets are datasets containing data collected during clinical trials, including data on patient outcomes and treatment effectiveness. They can be used to evaluate and optimize QoS in healthcare applications, such as by analyzing the impact of network latency on the accuracy and timeliness of data collection and analysis. Wearable device datasets are collected from wearable devices, such as fitness trackers and smart watches. They can be used to evaluate and optimize QoS in healthcare applications, such as by investigating the influence of network latency on the correctness and timeliness of data collection and analysis.

Numerous simulation technologies can be employed in applications for smart healthcare. Several scenarios were found in recent literature. A popular programming language used to create applications for smart healthcare is Java. Platform independence, security features, and the capacity to process enormous sizes of data are some of the characteristics of Java that make it suitable for healthcare applications. Java-based healthcare applications can be used to gather, store, and analyze patient data and can run on a range of devices, including smartphones, tablets, and laptops. Medical imaging software, telemedicine platforms, and electronic health record systems are a few examples of Java-based healthcare applications [46,59]. Healthcare applications for the numerical computing environment and programming language MATLAB (Matrix Laboratory) include image and signal processing, data analysis, and method development. MATLAB can be used in smart healthcare to create and analyze medical pictures, separate characteristics from signals, create prediction models, and create control schemes for medical equipment. To facilitate more complex analysis and decision-making, MATLAB can also be combined with other tools and libraries, such as those for machine learning and deep learning. Image-guided surgery, computer-assisted diagnostics, and patient monitoring are a few examples of smart healthcare applications that make use of MATLAB [47,50,52,56]. C++ is a sophisticated programming language that is popular in many industries, including in applications of smart healthcare. It is frequently employed in the creation of medical software, including clinical decision support systems, medical imaging software, and electronic health record systems. Additionally, C++ is used to create embedded systems and gadgets, such as wearable tech and medical sensors that may gather and transmit patient data for tracking and analysis. Additionally, C++ is frequently used to create simulations and other kinds of software for medical research. It is a preferred option for creating complicated and resource-intensive healthcare applications because of its flexibility and performance advantages, as mentioned in [51,58]. Healthcare applications can benefit from the usage of TensorFlow, a potent machine learning software framework that is available for free and open source. Predictive modeling, natural language processing, and image and signal analysis are some of the activities that TensorFlow can be used for in the healthcare industry [59]. The fields of complexity science and systems thinking frequently use the programming language and modeling environment known as NetLogo. It can be used to build simulations of intricate systems, such as those in healthcare. To simulate the effects of various treatment options on a patient’s health outcomes, for instance, or to model the transmission of a disease within a population, NetLogo can be employed. Additionally, the visualization features of NetLogo [70] can be utilized to provide interactive displays of medical data, which can be helpful for both researchers and practitioners. CloudSim [71] is a simulation tool used to model and evaluate cloud computing systems and services. In the context of smart healthcare, CloudSim can be used to simulate and analyze the performance of various healthcare systems that are based on cloud computing, such as electronic health record systems, telemedicine systems, and medical imaging systems. By simulating these systems in a controlled environment, researchers and developers can identify potential bottlenecks and optimize the performance of the systems before they are deployed in real-world settings [57]. Additionally, CloudSim can be used to evaluate the cost-effectiveness and scalability of different cloud-based healthcare solutions. Table 5 lists numerous QoS aware technologies, techniques, and datasets that are currently being used in healthcare applications.

### 7.5. Response to Research Question 5

QoS optimization in smart healthcare applications can be challenging due to several factors [72]. Major obstacles include network congestion, interoperability issues, real-time demand, and financial constraints. When too many users or devices attempt to access the same network at once, network congestion can occur, resulting in delayed or unstable connectivity. In healthcare, where many systems and devices may need to exchange information, interoperability refers to the capacity of various systems and equipment to operate together seamlessly. Real-time demands refer to the demand for very low latency in specific healthcare applications, such as remote surgery or monitoring critical patients. The cost of adopting and sustaining QoS might be a significant obstacle.

## 8. Challenges

As was stated in the response to research question No. 5, implementing SHAs involves a number of challenges. In the following section, these challenges are described in more detail with possible solutions.

### 8.1. Challenge 1

When improving healthcare apps, the cost must be considered. Cost is the amount of money spent on providing healthcare services while maintaining a standard of care given to patients. One solution for cost optimization in QoS in smart healthcare applications is to use data analytics and ML practices to detect patterns and trends in patient data and use this knowledge to make more informed decisions regarding resource allocation. Designing more efficient treatment protocols, identifying high-risk patients who may require more frequent monitoring, and optimizing session timing are some examples of how to achieve this. Another option to reduce the need for in-person visits and save healthcare costs is to use telemedicine and remote monitoring technologies [73]. Additionally, by reducing the number of people in need of medical care, the introduction of preventative measures and health awareness campaigns can help reduce costs.

### 8.2. Challenge 2

Security and privacy must be considered while implementing quality of service (QoS) optimization in smart healthcare systems. Sensitive patient data should be protected from unauthorized access and hackers using security measures like encryption and authentication. One can limit who has access to patient information by using access restrictions. Privacy is a significant problem in smart healthcare applications due to the sensitivity of personal health information. The de-identification of patient data, safe data storage, and strict access controls are all actions that can be taken to protect privacy. Healthcare organizations must also abide by appropriate rules and regulations, such as HIPAA in the US, to ensure that patient privacy is protected [74]. Owing to the blockchain’s decentralized structure, healthcare providers may securely share patient data while also guaranteeing that the data is impregnable and cannot be changed without the network’s consent. Additionally, smart contracts can automate several processes and ensure that only those with the right authorization can access data [75]. This has the potential to greatly improve healthcare delivery’s effectiveness and precision, which would ultimately benefit patients.

### 8.3. Challenge 3

Smart healthcare devices may generate a high volume of data, which can lead to network congestion and result in delays in data transmission [76]. There are many techniques to handle network congestion in smart healthcare applications in the context of QoS optimization. By utilizing cloud-based services and edge computing, network performance can be improved, and network load can be spread. Finally, having a solid network architecture may help guarantee that medical data is transmitted quickly and safely. One example is 5G networks [8,77].

### 8.4. Challenge 4

Smart healthcare devices from different manufacturers may not be able to communicate with each other, which can make it difficult to optimize QoS across the entire system. One approach to achieving interoperability in smart healthcare applications in the context of QoS improvement is to use a standard for data sharing, such as HL7 FHIR (fast healthcare interoperability resources) [78]. This standard makes it easy to transfer data between different platforms and systems while guaranteeing that the data is accurate and current. Making sure that data is consistently formatted and easy for different systems to interpret can also be accomplished with the use of a standard data model, such as the one provided by HL7 FHIR. Another approach is to implement a distributed architecture, which would allow for decentralized communication between various services, lessen dependency on a central hub, and boost robustness and scalability. The use of reliable and secure communication protocols, such as MQTT, CoAP, etc., could be an additional solution [79]. Regarding high throughput, low power consumption, and short latency, these protocols are designed to maximize service quality. Overall, building interoperability in smart healthcare applications while enhancing QoS requires the use of standardized data interchange formats and protocols as well as the creation of a robust and adaptable design that can adapt to the changing requirements of the healthcare system.

### 8.5. Challenge 5

Some smart healthcare applications have strict real-time requirements, such as in the case of remote monitoring and telemedicine, which can make QoS optimization challenging. Real-time constraints in smart healthcare systems can be met by applying QoS optimization techniques. These techniques include priority-based scheduling, efficient bandwidth and resource allocation, and the application of network protocols that facilitate real-time communication [80]. The system can also be made more responsive and able to meet real-time demands by using edge computing and a distributed design. It is also vital to assess and monitor the system’s performance to make sure that the goal QoS is being met and to adjust as needed. It is important for healthcare organizations to carefully consider these trade-offs and limitations when selecting and implementing QoS optimization methods to ensure that they meet the needs of the organization and its users [81].

In addition, authors in [82,83,84,85,86,87,88] presented several interesting and emerging research challenges related to the QoS, medical QoS, and QoE and then proposed various frameworks, methods, and algorithms. The effective and adaptive QoS optimization methods are proposed in [89,90,91,92,93]. The AI- and ML-driven classification and regression methods are the key role players in medical QoS and end-user perception analysis [94,95,96,97,98].

## 9. Recommendations and Lessons Learned

We have this opportunity to impart some recommendations from this extensive activity through SLR development and responding to the research questions. It became clear throughout the examination of the presented literature that smart healthcare is an interdisciplinary subject with roots in social and political studies in addition to health and engineering disciplines [99]. For instance, a patient should be allowed to decide whether to receive treatment from a real doctor or from an ML-based algorithm, and they should be fully informed about how and where their medical data will be utilized. From a technical standpoint, algorithms should be thoroughly examined and free of bias because otherwise, it could result in erroneous diagnoses [100]. Additionally, patients require accurate diagnoses, and emotional, and psychological support from the doctor, which may not be acceptable to the patient if a machine takes the doctor’s role for most of the doctor’s activities. The excessive adoption of technology may also interfere with the doctor–patient interaction, lowering expectations of care. The distribution of smart healthcare resources should initially go to underserved areas, and difficulties with the digital divide should be adequately handled, to maintain the quality of healthcare services [101]. To offer SHAs continual quality improvement, a research and review framework for partnerships at various levels should be established among healthcare stakeholders, academia, the research community, and the public.

The lessons which we learned can be summarized as follows:

- The most notable trend is to switch from conventional computing techniques to novel smart technologies to speed up the response time of SHAs.
- The most efficient method of computation is distributed since it is flexible and reduces delays.
- Due to their natural capacity to process enormous data contents, ML and DL algorithms are ideal for analyzing the health data that is currently available.
- Most readily accessible datasets are extremely general and have constrained access for researchers.
- Most of the evaluated literature concentrated on broad QoS issues, whereas SHA adoption requires more specialized research on its many practices and methodologies.

In summary, our research on QoS optimization in SHAs provided important new information about QoS-conscious techniques and practices. Adopting smart technologies, concentrating on AI techniques in SHAs, and enhancing QoS are some of the key lessons acquired. These lessons highlight the value of prompt responses and minimizing delays in SHAs. Our recommendations concentrate on the implementation of AI techniques with patients’ permission and the prioritization of resource distribution, with an emphasis on underserved areas.

## 10. Conclusions & Future Work

QoS optimization in smart healthcare applications is the process of ensuring that the efficacy and dependability of healthcare services meet or exceed that of patients and healthcare professionals. To do this, several methods can be used, for instance, resource allocation, network optimization, and the prioritization of critical tasks. Enhancing the durability of wireless networks to reduce delay and increase the efficiency of remote patient monitoring systems. Allocating computer power to ensure clinical decision support systems have the necessary processing power to provide accurate and timely guidance. The major goal of QoS optimization in smart healthcare apps is to increase the efficacy and quality of healthcare services. Our future work on QoS optimization in smart healthcare applications will evaluate how, by using blockchain technology, it is possible to store patient data securely, prevent tampering with it, and enable secure information flow between healthcare providers. Secondly, it will consider integrating 5G technology to support high-bandwidth applications like telemedicine, remote surgery, and real-time monitoring. Moreover, it will establish new QoS metrics that consider the unique requirements of smart healthcare applications, such as patient privacy and confidentiality.

## Figures and Tables

**Table 1:** Link strength analysis among IoT, ML, and cloud computing.

Links	Strength	Information
IoT ↔ ML	strong	more research articles published
IoT ↔ Cloud Computing	strong	more research articles published
Cloud Computing ↔ ML	weak	fewer research articles published

**Table 2:** List of Data Sources.

S.No.	Name	Web Link
1	IEEE Xplore Digital Library	www.ieeexplore.ieee.org (accessed on 10 January 2023)
2	ScienceDirect	www.sciencedirect.com (accessed on 17 January 2023)
3	Google Scholar	www.scholar.google.com (accessed on 30 January 2023)

**Table 3:** Comparative evaluation of the examined publications using basic SLR criteria.

Ref.	Review Type	Main Topic	Year	Covered	Pre-SLR	Criteria	Papers Screened	RQs	QoS
[35]	SLR	IoMT	2022	2005–2020	NA	Citation Impact factor ≥ 1	135	Not clear	NA
[36]	SLR	IoT	2022	2015–2022	NA	Keywords	106	Reasons constraints solutions	Cost
[37]	SLR	IoT	2022	2015–2021	NA	Quality assessment score	22	IoT adoption	NA
[38]	SLR	IoT AI	2022	2016–2021	NA	Citation count impact factor journals	104	E-Cardiac	NA
[39]	SLR	IoT	2021	NA	NA	Keywords	81	Edge computing Blockchain	NA
[40]	Survey	Blockchain	2019	NA	NA	Keywords	NA	NA	NA
[41]	PRISMA	ML	2022	2016–2021	NA	Keywords	50	Smart Technologies	Network
[42]	PRISMA	Smart Healthcare	2022	2015–2021	NA	Geographical	26	Adoption challenges	NA
Our	Hybrid	QoS Optimization	2023	2018–2023	Mentioned	Recent keywords’ frequency	60	Optimization issues	Optimization

**Table 4:** Simulation models, QoS metrics, and sensor-based trends in filtered articles.

Ref.	Model	QoS Metric	Sensors
[46]	Matching based model	Latency, energy consumption	IoT
[47]	Deep Reinforcement Learning (DRL) model	Energy consumption and latency	Mobile sensors
[48]	DL models	Energy consumption and latency	IoT
[49]	Monte Carlo	Throughput and energy efficiency	Medical and motion
[50]	Hybrid fog–cloud of offloading (HFCO)	delay	IoT
[51]	Confident information coverage (CIC) model	Energy consumption	Mobile sensors
[52]	Cluster-based hierarchical approach	Energy consumption	Smart Sensors
[53]	Cloud Based Models	Energy consumption	N/A
[54]	Agent-based modeling and Ontology	Overall QoS	Body sensors
[55]	Secure human-centric mobility-aware (SHM) model	Throughput and latency	CPS sensors
[56]	Grey Filter Bayesian Convolution Neural Network (GFB-CNN)	Delay and latency	Smart IoT sensors
[58]	Clustered federated learning (CFL) model	Latency	Smart IoT sensors
[59]	Network model	Latency	IoT sensors

**Table 5:** Selected papers to show different technologies, techniques, tools, and datasets.

Ref.	Technologies	Methods	Simulation Tools	Datasets
[55]	Resource allocation	Bandwidth allocation, QoE	Java platform	Wearable device datasets
[46]	Machine learning predictive analytics	Prioritization	MATLAB	EEG dataset
[47]	Machine learning predictive analytics	Load balancing	Wireless brain monitoring system	N/A
[49]	Resource allocation	Prioritization	N/A	Wearable device datasets
[50]	Machine learning predictive analytics	Load balancing, QoE	MATLAB	Electronic medical records (EMR) dataset
[48]	Traffic management	Error correction	MATLAB, C++/C#	Wearable device datasets
[51]	Machine learning predictive analytics	Load balancing	Not mentioned	Wearable device datasets
[52]	Resource allocation	Load balancing	MATLAB	Electronic medical records (EMR) dataset
[53]	Resource allocation	Load balancing	NA	Wearable device datasets
[54]	Machine learning predictive analytics	QoE measurement	NetLogo	Electronic medical records (EMR) dataset mHealth (mobile health)
[56]	Quality of experience	Prioritization	CloudSim	Wearable device datasets
[64]	Security tools	QoE measurement	C++/Java	Electronic medical records (EMR) dataset
[59]	Traffic management	Traffic shaping	TensorFlow	Electronic medical records (EMR) dataset X-ray and ultrasound
